# Pregnant Women’s Perceptions of Harms and Benefits of Mental Health Screening

**DOI:** 10.1371/journal.pone.0145189

**Published:** 2015-12-22

**Authors:** Dawn Kingston, Marie-Paule Austin, Sheila W. McDonald, Lydia Vermeyden, Maureen Heaman, Kathleen Hegadoren, Gerri Lasiuk, Joshua Kingston, Wendy Sword, Karly Jarema, Sander Veldhuyzen van Zanten, Sarah D. McDonald, Anne Biringer

**Affiliations:** 1 Faculty of Nursing, Department of Obstetrics and Gynecology, University of Alberta, Edmonton, Alberta, Canada; 2 St John of God Chair Perinatal & Women’s Mental Health, School of Psychiatry, University of New South Wales, Sydney, Australia; 3 Population, Public, and Aboriginal Health, Alberta Health Services, Calgary, Alberta, Canada; 4 Faculty of Nursing, University of Alberta, Edmonton, Alberta, Canada; 5 College of Nursing, Faculty of Health Sciences, University of Manitoba, Winnipeg, Manitoba, Canada; 6 Faculty of Nursing, University of Saskatchewan, Regina, Saskatchewan, Canada; 7 Faculty of Science (Computer Science), MacEwan University, Edmonton, Alberta, Canada; 8 School of Nursing, Faculty of Health Sciences, University of Ottawa, Ottawa, Ontario, Canada; 9 Division of Gastroenterology, University of Alberta, Edmonton, Alberta, Canada; 10 Division of Maternal-Fetal Medicine, Departments of Obstetrics & Gynecology, Radiology, and Clinical, Epidemiology & Biostatistics McMaster University, Hamilton, Ontario, Canada; 11 Department of Family and Community Medicine, University of Toronto, Ada and Slaight Family Director of Maternity Care, Ray D Wolfe Department of Family Medicine, Mount Sinai Hospital, Toronto, Ontario, Canada; Medical University of Vienna, AUSTRIA

## Abstract

**Background:**

A widely held concern of screening is that its psychological harms may outweigh the benefits of early detection and treatment. This study describes pregnant women's perceptions of possible harms and benefits of mental health screening and factors associated with identifying screening as harmful or beneficial.

**Methods:**

This study analyzed a subgroup of women who had undergone formal or informal mental health screening from our larger multi-site, cross-sectional study. Pregnant women >16 years of age who spoke/read English were recruited (May-December 2013) from prenatal classes and maternity clinics in Alberta, Canada. Descriptive statistics were generated to summarize harms and benefits of screening and multivariable logistic regression identified factors associated with reporting at least one harm or affirming screening as a positive experience (January-December 2014).

**Results:**

Overall study participation rate was 92% (N = 460/500). Among women screened for mental health concerns (n = 238), 63% viewed screening as positive, 69% were glad to be asked, and 87% took it as evidence their provider cared about them. Only one woman identified screening as a negative experience. Of the 6 harms, none was endorsed by >7% of women, with embarrassment being most cited. Women who were very comfortable (vs somewhat/not comfortable) with screening were more likely to report it as a positive experience.

**Limitations:**

Women were largely Caucasian, well-educated, partnered women; thus, findings may not be generalizable to women with socioeconomic risk.

**Conclusions:**

Most women perceived prenatal mental health screening as having high benefit and low harm. These findings dispel popular concerns that mental health screening is psychologically harmful.

## Background

Despite rates that are similar to or exceed the most prevalent pregnancy-related complications screened for and treated during pregnancy [1–4], mental health is not comprehensively assessed as a component of routine prenatal care in Canada and the U.S. However, without early detection and treatment, the risk of chronicity is high. Recent epidemiologic evidence from two longitudinal birth cohorts suggests that 30–40% of women with prenatal depression continue to experience symptoms at 4 and 5 years postpartum [5,6]. In addition to highlighting the high rates of persistent depression beginning in pregnancy, these studies also revealed that chronic depression is associated with sub-optimal development in 19% of children of mothers with sub-clinical depressive symptoms and 24% of those whose mothers had clinical depressive symptoms (versus 7% in the ‘no depression’ group) [5]. Emerging findings from a small feasibility trial are promising in suggesting that antenatal psychological therapy may be effective for the reduction of antenatal anxiety symptoms, the rate of postpartum depression, and more optimal infant development outcomes, compared with the control group. [7] Thus, early identification and treatment of prenatal mental health problems may have substantial long-term benefits for maternal and child wellbeing.

A main barrier to early identification and subsequent treatment of prenatal mental health problems is the lack of routine prenatal mental health screening. While supported by international organizations [8–10], and most recently the American College of Obstetricians and Gynecologists [11], mental health screening has not been widely adopted within the context of mainstream prenatal care. One of the barriers to implementation is providers’ and policy-makers’ concern that a majority of women will experience psychological harm as a result of the screening process (e.g., stigma; false-positives). While a few qualitative studies suggest that some women have negative experiences during prenatal and postnatal mental health screening [12,13], other studies report general acceptability [14–18], including among women with high depressive symptoms [16]. Indeed, existing evidence for acute and long-term psychological harm of screening is poor [19,20], and much of this research has been generated within the context of cancer screening [21,22]. Thus, there is a need for rigorous evidence concerning the relative benefits and harms of mental health screening during pregnancy. The purposes of this study were to describe pregnant women’s perceptions of the benefits and harms of mental health screening and factors associated with identifying screening as harmful or beneficial (including sociodemographics, previous mental health diagnoses, type of provider, and barriers to disclosure).

## Methods

### Study Design and Inclusion Criteria

For this cross-sectional, multi-site study, pregnant women were consecutively recruited between May and November, 2013 from the two community hospital-based prenatal classes and five maternity clinics in Alberta, Canada. Pregnant women were eligible if they: 1) were >16 years of age; and 2) could speak/read English. The recruitment sites served a socioeconomically diverse patient population. Recruitment and study procedures have been described previously [23]. Approval was granted by the University of Alberta Research Ethics Board.

### Data Collection

Detailed description and development of the *Barriers and Facilitators of Mental Health Screening Questionnaire* have been reported previously [23]. Briefly, the 63-item self-report questionnaire was designed to identify barriers to and facilitators of screening and responses to mental health screening in pregnancy, including perceived harms and benefits. We have previously reported on results related to women’s acceptance of screening and preferences for method of screening [23] and barriers and facilitators of screening [24]. A computer tablet-based version of the questionnaire, built and tested by the Women’s and Children’s Health Research Institute (University of Alberta), was utilized for data collection. Once participants provided their written, informed consent and then submitted their responses to the survey, data were encrypted and transferred to the Faculty of Medicine and Dentistry at University of Alberta. All participants were 16+ years of age and gave written, informed consent electronically which the Research Ethics Board of the University of Alberta approved for consent procedure.

### Main Outcomes

The main outcomes of this sub-analysis were women’s perceptions of the benefits and harms of mental health screening during pregnancy. A statement introducing the list of harms (n = 6) and benefits (n = 3) indicated, “**There are many different ways that care providers may use to ask you about your emotional health (e.g., your mood or anxiety) during pregnancy…..How would you describe your experience of being asked about your emotional health?”** and asked women to respond ‘yes’ or ‘no’ to each specific statement of benefit or harm. Specific benefits that we assessed were: felt provider cared; glad to be asked; and was a positive experience. Items related to harms were: felt embarrassed; did not know why my provider asked certain questions; was a negative experience; felt worried about what would happen with my information; the way questions were asked made me uncomfortable; and the questions made me uncomfortable.

### Sample Size Estimation and Analysis

This study utilized the sub-set of participants from the larger multi-site, cross-sectional study who indicated that their provider had formally (e.g., using a screening tool) or informally (e.g., verbal inquiry) screened them for mental health concerns during pregnancy (n = 238). Sample size estimation was based on the intent to use multivariable regression to identify factors associated with harms and benefits (100 + 8m, where m = number of predictors) [25]. We conservatively estimated 15 independent variables, thus requiring a minimum sample size of 220.

Descriptive statistics (proportions, means, standard deviations) were generated. For the multivariable logistic regression analysis of factors associated with perceived harms, we constructed the outcome variable ‘identified one or more harms of screening’ because fewer than 7% of women endorsed any single harm. For the model describing factors associated with benefits of screening, we used women’s overall assessment, ‘It was a positive experience’ (yes versus no) as the outcome. We could not use the item, ‘It was a negative experience’ (yes versus no) as originally planned, because only one woman responded affirmatively. We estimated unadjusted odds ratios for the association between each independent variable and outcome. In particular, we were interested in associations between the outcomes and demographic variables, previous diagnosis or treatment for a mental health problem, type of provider, comfort with the screening process, two stigma-related factors (worry about being seen as a bad mother; not wanting to be seen by provider as anxious or depressed), and six perceived barriers to screening (e.g., worry that provider would not think mental health concerns were important; provider does not have time to talk; I feel I could handle mental health problems on my own; significant other has told me my emotions are normal for pregnancy and not to worry; worry about being put on antidepressants; would rather discuss emotional concerns with significant others). Variables associated with outcomes at p<0.20 were included in the multivariable logistic regression analyses with statistical significance for final models set at p<.05. All variables were adjusted for each other. Correlations among independent variables that met criteria for model entry were generated to assess for multicollinearity (r>0.4). Adjusted odds ratios and 95% confidence intervals were calculated. Statistical analyses were conducted using SPSS version 21 (SPSS IBM, New York, USA). Missing data were minimized by using the online questionnaire that required women to respond before proceeding to subsequent questions. All analyses were conducted January-December 2014.

## Results

The overall study participation rate was 92% (N = 460/500). Of the 238 women in the larger sample who had been formally or informally screened for mental health concerns (51.7%; 238/460), over 80% were ≥25 years of age (M = 29.0, SD 4.4), had some or completed post-secondary education, and were Caucasian (Table 1). Mean gestational age was 30 weeks (SD 5.4). One-quarter of women had previously been diagnosed with a mental health problem. No significant differences were found between the sub-group of women who had been screened and the overall sample (data not shown).

**Table 1:** Sample characteristics of pregnant women responding to Barriers and Facilitators of Mental Health Screening Questionnaire in Alberta, Canada (N = 238).

Variables	N	%
Maternal age at time of interview		
≥25 years	192	81.0
≤24 years	45	19.0
Maternal highest level of education completed		
Some or completed post-secondary	193	81.4
≤High school	44	18.6
Annual household income		
<$40,000	30	12.7
≥$40,000	207	87.3
Marital status		
Married/Common-Law	211	89.0
Other (Single, divorced, widowed)	26	11.0
Ethnicity		
Non-Caucasian*	46	19.4
Caucasian	191	80.6
Born in Canada		
Yes	199	84.0
No	38	16.0
Been pregnant before		
Yes	97	40.9
No	140	59.1
Diagnosed with depression or had depressive symptoms		
Yes, formally diagnosed	58	24.5
No, never formally diagnosed; never experienced symptoms	114	48.1
No, never formally diagnosed; experienced symptoms	65	27.4
Treated for depression, anxiety, or any emotional concern		
Yes	65	27.3
No	173	72.7
Most care in pregnancy provided by…		
Obstetrician	119	50.0
Family doctor	104	43.7
Other (midwife, nurse)	15	6.3

*Non-Caucasian status included options of Aboriginal, Arab/West Asian, Black, Chinese, Filipino, Japanese, Korean, South Asian, Latin American, South East Asian, Other.

In response to the question, ‘How would you describe the experience of being asked about your mental health’, 63% percent of women indicated ‘It was positive’ with one woman reporting ‘It was negative’ (Table 2). Over three-quarters of women (78%) indicated that a benefit of screening was that it made them feel their provider cared about them and 69% felt glad to be asked. Seventeen percent (n = 40) of the sample identified at least one harm and 2% (n = 5) reported 2 or more. Each of the harms related to screening that we assessed (Table 2) was endorsed by less than 7% of women. The most common harm identified was feeling embarrassed (n = 16, 6.7%) (Table 2).

**Table 2:** Description of responses to screening identified by pregnant women who underwent prenatal mental health screening in the Barriers and Facilitators of Mental Health Screening Questionnaire in Alberta, Canada (N = 238).

	N	%
**Response to Screening: Benefits**		
Felt provider cared		
Yes	186	78.2
No	52	21.8
Glad to be asked		
Yes	164	68.9
No	74	31.1
Was a positive experience		
Yes	150	63.0
No	88	37.0
**Response to Screening: Harms**		
Felt embarrassed		
Yes	16	6.7
No	222	93.3
Did not know why he/she asked certain questions		
Yes	9	3.8
No	229	96.2
Was a negative experience		
Yes	1	0.4
No	237	99.6
Felt worried about what would happen with my information		
Yes	13	5.5
No	225	94.5
Way questions were asked made me uncomfortable		
Yes	4	1.7
No	234	98.3
Questions made me uncomfortable		
Yes	4	1.7
No	234	98.3
Woman identified 1 or more harms		
Yes	40	16.8
No	198	83.2
Woman identified 2 or more harms		
Yes	5	2.1
No	233	97.9
Woman identified 3 or more harms		
Yes	2	0.8
No	236	99.2
Woman identified 4 or more harms		
Yes	0	0.0
No	238	100.0

### Benefits of screening

In the multivariable model examining factors associated with identifying screening as a positive experience (Table 3), the only significant finding was that women who were very comfortable with mental health screening (85.3%) had over twice the odds of reporting screening as positive (AOR 2.43, 95% CI 1.18–4.98), compared with those who were somewhat or not at all comfortable with screening (14.7%).

**Table 3:** Factors associated with reporting mental health screening as a positive experience among pregnant women who had undergone mental health screening in Alberta, Canada (as reported in the Barriers and Facilitators of Mental Health Screening Questionnaire) (N = 238).

Independent Variable	Had a positive experience			
	Yesn (%)	Non (%)	UOR(95% CI)	AOR (95% CI)
Age				
≥25 years	128 (85.9)	64 (72.7)	2.29 (1.18–4.41)**	1.95 (.90–4.25)
≤24 years	21 (14.1)	24 (27.3)	1.00	1.00
Highest level of education				
Some or all postsecondary education	128 (85.9)	65 (73.9)	2.16 (1.11–4.18)**	1.51 (.67–3.39)
High school or less	21 (14.1)	23 (26.1)	1.00	1.00
Household income				
<$40,000	10 (6.7)	20 (22.7)	.25 (.11-.55)**	.41 (.14–1.20)
≥$40000	139 (93.3)	68 (77.3)	1.00	1.00
Marital status				
Married or living common-law	138 (92.6)	73 (83.0)	2.58 (1.13–5.90)**	1.13 (.37–3.41)
Other (single, widowed)	11 (7.4)	15 (17.0)	1.00	1.00
Ethnicity				
Non-Caucasian	18 (12.1)	28 (31.8)	.29 (.15-.57)**	.54 (.22–1.33)
Caucasian	131 (87.9)	60 (68.2)	1.00	1.00
Born in Canada				
Yes	130 (87.2)	69 (78.4)	1.88 (.94–3.79)*	1.17 (.45–3.01)
No	19 (12.8)	19 (21.6)	1.00	1.00
Ever been pregnant before				
Yes	64 (43.0)	33 (37.5)	1.26 (.73–2.15)	
No	85 (57.0)	55 (62.5)	1.00	
Ever diagnosed with depression, anxiety, or any other kind of emotional concern by a healthcare provider				
Yes	36 (24.2)	22 (25.0)	.96 (.52–1.76)	
No	113 (75.8)	66 (75.0)	1.00	
Ever treated for depression, anxiety, or any other kind of emotional concern				
Yes	38 (25.3)	27 (30.7)	.77 (.43–1.37)	
No	112 (74.7)	61 (69.3)	1.00	
Type of provider				
Family doctor	66 (44.0)	38 (43.2)	1.10 (.63–1.90)	1.10 (.60–2.01)
Other (midwife, nurse)	6 (4.0)	9 (10.2)	2.85 (.95–8.57)*	1.58 (.42–5.91)
Obstetrician	78 (52.0)	41 (46.6)	1.00	1.00
Would worry provider would not think concerns are important				
Agree	20 (13.3)	26 (29.5)	.37 (.19-.71)**	.45 (.20–1.01)
Disagree	130 (86.7)	62 (70.5)	1.00	1.00
Provider doesn’t have time to talk				
Agree	39 (26.0)	31 (35.2)	.65 (.37–1.14)	
Disagree	111 (74.0)	57 (64.8)	1.00	
Feel that I could handle my mood on my own				
Agree	103 (68.7)	67 (76.1)	.69 (.38–1.25)	
Disagree	47 (31.3)	21 (23.9)	1.00	
Worry I would be seen as a bad mother				
Agree	29 (19.3)	27 (30.7)	.54 (.30-.99)**	.88 (.41–1.89)
Disagree	121 (80.7)	61 (69.3)	1.00	1.00
Would not want to be seen as depressed or anxious by provider				
Agree	58 (38.7)	41 (46.6)	.72 (.42–1.23)	
Disagree	92 (61.3)	47 (53.4)	1.00	
Partner, friend, or family have told me that my emotional swings are ‘normal’ and not to worry				
Agree	116 (77.3)	61 (69.3)	1.51 (.84–2.73)	
Disagree	34 (22.7)	27 (30.7)	1.00	
Worry about being put on antidepressants				
Agree	60 (40.0)	46 (52.3)	.61 (.36–1.04)*	.79 (.43–1.47)
Disagree	90 (60.0)	42 (47.7)	1.00	1.00
Would rather discuss emotional concerns with partner, friends, or family				
Agree	96 (64.0)	59 (67.0)	.87 (.50–1.52)	
Disagree	54 (36.0)	29 (33.0)	1.00	
Felt comfortable with screening				
Very Comfortable	128 (85.3)	60 (69.0)	2.62 (1.38–4.97)**	2.43 (1.18–4.98)**
Somewhat comfortable, somewhat uncomfortable, or very uncomfortable	22 (14.7)	27 (31.0)	1.00	1.00

*p<0.20 in univariable analysis, therefore met criteria for entry to multivariable models.

**p<0.05

### Harms of screening

In the multivariable model with the outcome ‘identified one or more harms of screening’, no variables that we assessed were significant, including demographics, type of provider, barriers or stigma-related factors (Table 4). While a previous diagnosis of a mental health problem was significantly related to identifying at least one harm of screening in the univariable analysis (37.5% vs 21.8%), it was not significant in the multivariable model.

**Table 4:** Factors associated with identifying one or more harms of mental health screening among pregnant women who had undergone mental health screening in Alberta, Canada (as reported in the *Barriers and Facilitators of Mental Health Screening Questionnaire*) (N = 238).

Independent Variable	Identified one or more harms of screening			
	Yesn (%)	Non (%)	UOR (95% CI)	AOR (95% CI)
Age				
≥25 years	32 (80.0)	160 (81.2)	.93 (.39–2.17)	
≤24 years	8 (20.0)	37 (18.8)	1.00	
Highest level of education				
Some or all postsecondary education	34 (85.0)	159 (80.7)	1.35 (.53–3.46)	
High school or less	6 (15.0)	38 (19.3)	1.00	
Household income				
<$40,000	5 (12.5)	25 (12.7)	.98 (.35–2.74)	
≥$40000	35 (87.5)	172 (87.3)	1.00	
Marital status				
Married or living common-law	33 (82.5)	178 (90.4)	.50 (.20–1.29)	
Other (single, widowed)	7 (17.5)	19 (9.6)	1.00	
Ethnicity				
Non-Caucasian	10 (25.0)	36 (18.3)	1.49 (.67–3.32)	
Caucasian	30 (75.0)	161 (81.7)	1.00	
Born in Canada				
Yes	34 (85.0)	165 (83.8)	1.10 (.43–2.83)	
No	6 (15.0)	32 (16.2)	1.00	
Ever been pregnant before				
Yes	20 (50.0)	77 (39.1)	1.56 (.79–3.08)	
No	20 (50.0)	120 (60.9)	1.00	
Ever diagnosed with depression, anxiety, or any other kind of emotional concern by a healthcare provider				
Yes	15 (37.5)	43 (21.8)	2.15 (1.04–4.43)**	2.05 (.96–4.39)
No	25 (62.5)	154 (78.2)	1.00	1.00
Ever treated for depression, anxiety, or any other kind of emotional concern				
Yes	15 (37.5)	50 (25.3)	1.78 (.87–3.63)	
No	25 (62.5)	148 (74.7)	1.00	
Type of provider				
Family doctor	15 (37.5)	89 (44.9)	1.20 (.58–2.48)	
Other (midwife, nurse)	5 (12.5)	10 (5.1)	.40 (.13–1.31)	
Obstetrician	20 (50.0)	99 (50.0)	1.00	
Would worry provider would not think concerns are important				
Agree	12 (30.0)	34 (17.2)	2.07 (.96–4.47)*	1.37 (.55–3.40)
Disagree	28 (70.0)	164 (82.8)	1.00	1.00
Provider doesn’t have time to talk				
Agree	14 (35.0)	56 (28.3)	1.37 (.67–2.80)	
Disagree	26 (65.0)	142 (71.7)	1.00	
Feel that I could handle my mood on my own				
Agree	31 (77.5)	139 (70.2)	1.46 (.66–3.26)	
Disagree	9 (22.5)	59 (29.8)	1.00	
Worry I would be seen as a bad mother				
Agree	15 (37.5)	41 (20.7)	2.30 (1.11–4.75)**	1.37 (.56–3.33)
Disagree	25 (62.5)	157 (79.3)	1.00	1.00
Would not want to be seen as depressed or anxious by provider				
Agree	23 (57.5)	76 (38.4)	2.17 (1.09–4.33)**	1.72 (.77–3.81)
Disagree	17 (42.5)	122 (61.6)	1.00	1.00
Partner, friend, or family have told me that my emotional swings are ‘normal’ and not to worry				
Agree	32 (80.0)	145 (73.2)	1.46 (.63–3.37)	
Disagree	8 (20.0)	53 (26.8)	1.00	
Worry about being put on antidepressants				
Agree	18 (45.0)	88 (44.4)	1.02 (.52–2.03)	
Disagree	22 (55.0)	110 (55.6)	1.00	
Would rather discuss emotional concerns with partner, friends, or family				
Agree	26 (65.0)	129 (65.2)	.99 (.49–2.03)	
Disagree	14 (35.0)	69 (34.8)	1.00	
Felt comfortable with screening				
Very Comfortable	25 (64.1)	163 (82.3)	.38 (.18-.81)**	.49 (.22–1.07)
Somewhat comfortable, somewhat uncomfortable, or very uncomfortable	14 (35.9)	35 (17.7)	1.00	1.00

*p<0.20 in univariable analysis, therefore met criteria for entry to multivariable models.

**p<0.05

Because the most common harm identified by women was feeling embarrassed, we sought to understand what characterized women who reported feeling embarrassed during screening. No variables had statistically significant associations, including demographics, history of diagnosis or treatment, type of provider, barriers of screening or either of the ‘stigma’ variables (worry I would be seen as a bad mother would not want to be seen as depressed or anxious by provider) (data not shown).

## Discussion

The current study reports on pregnant women’s perceived harms and benefits of mental health screening. To our knowledge, it is the first study to report quantitative data regarding harms and benefits of prenatal mental health screening. The majority of women identified screening as beneficial and reported that it made them feel that their provider cared. Only one woman reported that her experience of screening was negative. Each harm of screening that we assessed was endorsed by less than 7% of women, with the most common harm identified as feeling embarrassed. Demographics, type of provider, barriers and stigma-related factors were not related to benefits or harms of screening. Women who were very comfortable with mental health screening were more likely to identify screening as positive compared with those who were somewhat or not at all comfortable. No factors that we assessed were associated with endorsing one or more harms of screening.

The view of screening as a positive experience held by most women in this study stands in contrast to the perceptions held by some prenatal care providers that women view screening negatively [26]. While prenatal care providers cite women’s negative responses to screening as deterrents to implementing routine screening, including women’s unwillingness to discuss mental health, accept medication, receive counseling, or accept diagnoses [27–30], our findings do not support clinicians’ and policymakers’ perceptions of women’s views of screening as harmful. A more broadly held concern of screening and an argument that is frequently used against screening (albeit with minimal evidence) is the potential psychological harm that is inflicted on the patient through screening, including false-positive results [31]. However, the high proportion of women who report screening as a positive experience and the low proportion that report it as negative (<1%) or harmful (<7%) does not support this presupposition. Moreover, women’s responses in this study suggest that they were glad to be asked about their mental health, identifying a key benefit as enhancement of the provider-patient relationship. Reid et al. (1998) also reported that women felt that routine psychosocial screening promoted a closer relationship with their providers, allowing them to share ‘uncomfortable things’ [32].

Indeed, interest is growing in consumer perceptions of the relative benefits and harms of screening—more so in cancer care than mental health care. Recent studies exploring benefits of screening for cancer versus harms (when conceptualized as false-positives) report that 97% of women find the benefits of cancer screening outweigh the harms [33], that false-positive cancer screening results are not associated with significant psychological distress up to 3-years post-screening [19], and that tolerance for false-positive (i.e., overdetection) is high when weighed against the risk of non-detection [20]. Furthermore, pregnant women who participated in qualitative interviews conducted in follow-up to this study’s *Barriers and Facilitators* questionnaire (n = 50) indicated that they perceived the benefits of mental health screening during pregnancy as greater than the potential harms, even if it meant that they had a false-positive screen. These women agreed that they would prefer to have a false-positive result (i.e., screened as positive and then later told that they did not have depression/anxiety) than a false-negative result that called their concerns and suspicions about having a mental health problem into question (i.e., they suspected they had a problem but were told on screening that they did not).

### Benefit of Screening

Our findings revealed that demographic characteristics, being diagnosed or treated with a mental health problem, type of provider, barriers and stigma-related factors were not related to whether women viewed screening as a positive experience or not. This encouraging finding suggests that women universally view screening positively. The only factor associated with viewing screening as a positive experience or not was women’s comfort level with screening. In a previous study, we identified that the main contributor to women’s comfort with screening was whether they felt that they could be honest in disclosing mental health concerns with their provider [23]. Our previous research also suggests that pregnant women are most comfortable with screening when providers initiate the process (e.g., through routine prenatal mental health screening vs non-routine), and when more anonymous modes of screening are used (e.g., paper- and computer-based screening vs self-initiated face-to-face or telephone) [23]. Others have identified women’s lack of comfort as a deterrent to screening and suggest that comfort is more related to provider-oriented characteristics than to environmental factors [30]. Indeed, this research suggests that women are generally prepared and willing to disclose their mental health concerns. However, relational discomfort and providers’ deliberate decision not to facilitate disclosure of symptoms has potential to shift women’s conscious decision to one of non-disclosure [30]. As such, creating comfort by employing key low-resource strategies, such as establishing a relationship of trust, being aware of and eliminating stigmatizing behaviours (e.g., minimizing concerns) and using basic relationship skills (e.g., eye contact; listening) [30], are powerful approaches for optimizing the screening process and early detection. Indeed, as reported in this study by 78% of women, screening made them feel that their provider cared and in other studies [32], the very act of screening can have positive, ancillary benefits on the provider-patient relationship.

### Harms of Screening

The lack of association with demographic factors, being diagnosed or treated with a mental health problem, barriers or stigma-related factors and identifying harms of screening is reassuring in that no specific subgroups of women perceived screening as harmful or identified the most common harm of feeling embarrassed during screening. The stigma-related variables (worry what provider would think; worry about being seen as a bad mother) also were unrelated to embarrassment in both unadjusted and adjusted models (data not shown), which suggests that embarrassment in screening may not be due to stigma. Facilitators of screening (e.g., provider was sensitive and interested, knew that talking about mental health was a part of normal prenatal care, reassurance that other pregnant women have emotional problems) were also unrelated to whether women identified a harm of screening as embarrassment or not. Thus, while in a few qualitative studies some women have identified harms of screening (uncomfortable, intrusive) [13], our data suggest that this is the experience of a minority of women.

### Limitations

Over 80% of women in this sample were socioeconomically advantaged and Caucasian. Thus, while our findings did not reveal that less disadvantaged or non-Caucasian women perceived harms and benefits differently, this study should be replicated among vulnerable women, including sociodemographically disadvantaged and minority women. The findings related to harms of screening must be interpreted with caution because the number of women that identified harms (n = 40) and being embarrassed (n = 16) was low and this analysis may have been underpowered to detect an association. However, the number of variables that met criteria for entry into the final multivariable models for harms (n = 5) and embarrassment (n = 2) also was small but was within parameters for detecting significant associations if they existed at a power of 80%. Finally, although we asked whether women had “ever” been diagnosed or treated for depression, anxiety or any other form of emotional concern, we did not expressly ask women whether they currently suffered with these conditions.

## Conclusion

A central argument in the debate against screening is the concern that the harms exceed the benefits of screening. While this widely held position is founded on little evidence, results from this multisite study provide empiric evidence that the majority of pregnant women view screening as having high benefit and low harm. The finding that no demographic variables (including previous history of diagnosis or treatment for mental health problems) were associated with identifying harm in screening is positive in that it suggests that this view is not held by specific sub-groups of women. Thus, these findings may alleviate clinicians’ and policy-makers’ concerns regarding the negative impact of mental health screening on pregnant women.
